# Development of a monitoring instrument to assess the performance of the Swiss primary care system

**DOI:** 10.1186/s12913-017-2696-z

**Published:** 2017-11-29

**Authors:** Sonja T. Ebert, Valérie Pittet, Jacques Cornuz, Nicolas Senn

**Affiliations:** 1 Department of Ambulatory Care and Community Medicine, University of Lausanne, Bugnon 44, 1011 Lausanne, Lausanne, Switzerland; 2 Institute of Social and Preventive Medicine, University of Lausanne, Biopole 2, 1010 Lausanne, Switzerland; 3 Institute of Family Medicine, University of Lausanne, Lausanne, Switzerland

**Keywords:** Swiss, Primary care, Monitoring, Rand, Indicator, Performance, Effectiveness

## Abstract

**Background:**

The Swiss health system is customer-driven with fee-for-service paiement scheme and universal coverage. It is highly performing but expensive and health information systems are scarcely implemented. The Swiss Primary Care Active Monitoring (SPAM) program aims to develop an instrument able to describe the performance and effectiveness of the Swiss PC system.

**Methods:**

Based on a Literature review we developed a conceptual framework and selected indicators according to their ability to reflect the Swiss PC system. A two round modified RAND method with 24 inter−/national experts took place to select primary/secondary indicators (validity, clarity, agreement). A limited set of priority indicators was selected (importance, priority) in a third round.

**Results:**

A conceptual framework covering three domains (structure, process, outcome) subdivided into twelve sections (funding, access, organisation/ workflow of resources, (Para-)Medical training, management of knowledge, clinical−/interpersonal care, health status, satisfaction of PC providers/ consumers, equity) was generated.

365 indicators were pre-selected and 335 were finally retained. 56 were kept as priority indicators.- Among the remaining, 199 were identified as primary and 80 as secondary indicators. All domains and sections are represented.

**Conclusion:**

The development of the SPAM program allowed the construction of a consensual instrument in a traditionally unregulated health system through a modified RAND method. The selected 56 priority indicators render the SPAM instrument a comprehensive tool supporting a better understanding of the Swiss PC system’s performance and effectiveness as well as in identifying potential ways to improve quality of care. Further challenges will be to update indicators regularly and to assess validity and sensitivity-to-change over time.

**Electronic supplementary material:**

The online version of this article (10.1186/s12913-017-2696-z) contains supplementary material, which is available to authorized users.

## Background

The 2011 OECD Health report (Organisation for Economic Co-operation and Development) stated that the Swiss health care system, a unique customer-driven fee-for-service system with universal coverage, is high performing [1]. The International Health Policy Survey of the Commonwealth Fund 2013 showed a very high satisfaction rate of the population with more than ninety percents of the surveyed persons being satisfied or very satisfied with their PC physicians services. It is also viewed by health actors as excellent as 84% declared in a recent international survey to be satisfied or very satisfied [2]. However and paradoxically, health information seems is limited and few data are available [3], especially with regard to primary care (PC) [4]. Switzerland is considered to be an effective health system [3] and ranked second in the Commonwealth health systems performance report [5], but ranked also among the top 5 most expensive health systems, spending 11.4% of the Gross domestic product (GDP) on health in 2009 (OECD average of 9.5% of GDP). In a rapidly evolving and more complex health system (increase of an ageing (patient- and provider-) population and a rise of chronic and multimorbid conditions), a PC model mostly centered on GP activities might not be sustainable considering the current demography of physicians [1].

In 2009, the Netherlands Institute for Health Services Research (Nivel) launched the PHAMEU collaboration (Primary Health Care Activity Monitor for Europe) [6]. The purpose of this project was to develop a PC monitoring instrument that could be used in Europe and allows drawing comparison between countries. Following the model developed by Donabedian [7] the PHAMEU tool is organized classically into 3 main domains: structure, process and outcomes. It is composed of almost 100 indicators grouped into nine main sections: governance, economics, workforce, access, comprehensiveness, continuity, coordination, quality and efficiency. The department of ambulatory care and community medicine in Lausanne (Policlinique Médicale Universitaire, PMU), Switzerland was involved in the PHAMEU project and coordinated it for Switzerland. The PHAMEU study [6, 8], confirmed that a limited amount of data was available in Switzerland that accurately describes the functioning and performances of PC. Indeed, almost half of the indicators (*N* = 45/91) could not be built on existing data and relied exclusively on experts’ opinion [4].

In 2003, Marshall et al. [9] highlighted that the comparison of health care systems of different countries through indicators should be treated with caution. Other studies showed that indicator-convertibility e.g. between the US and the UK or the US and the Netherlands vary around 56.3% and 67% in the average [9–11]. Furthermore, an instrument such as the PHAMEU tool is well designed to compare PC between countries, but can only partially integrate local contextual specificities. Finally, the implicitly formulated indicators are often too generic to accurately describe changes over time. Based on these observations, developing a comprehensive instrument with an elevated appropriateness to the Swiss context and ability to describe the functioning of PC in Switzerland appears necessary. Moreover, even if key regulations elements are managed at a national level (unique board certification, global governance, compulsory insurance system,…), the organization of the provision of care (from hospitals to community care) is highly fragmented in Switzerland as it is left to the regional health authorities (26 cantons), what represents an additional challenge for the monitoring of health care at national level and data collection.

### Objective of the present study

This study aims to describe the development of a monitoring instrument for the measurement of performance and effectiveness of the Swiss PC system, the SPAM (Swiss Primary Health Care Active Monitoring) program. The program aims to evaluate PHC activities over time and how it de facto operates at a population level [12]. It should be able to provide synthetic information to all actors of PC (policy makers, health care providers, public health authorities and academic institutions) by means of explicitly formulated indicators via a modified RAND consensus method.

## Design and methods

### Conceptual framework

**Fig. 1:**
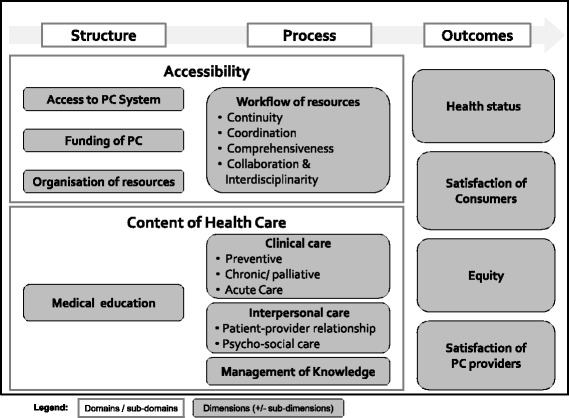
The SPAM conceptual framework

In order to provide a structure for the identification and organization of indicators, an operational framework was elaborated. The model of PHAMEU [6], itself strongly inspired by Donabedian, [13] was initially selected as a basis. The rather similar model developed by Campbell et al. [14] in the United Kingdom was also used to complete the initial model. The main addition linked to this model was to provide a more detailed description of the performance domains such as effectiveness or accessibility. The initial model was then discussed with opinion leaders in Public Health, Epidemiology and General Internal Medicine (Practitioner and Researchers) of the Institute of Social and Preventive Medicine, the Institute of General Medicine, the Department of Ambulatory Care and Community Medicine from Lausanne University and the Swiss Health Observatory. These experts critically reviewed the initial model in order to come up with a coherent conceptual framework with the perspective of monitoring the Swiss PHC system. Besides adapting several dimensions to reflect specific objectives of the project, the framework was finalized in consideration to the above mentioned specialists’ comments. Finally, the conceptual framework was submitted for review to the SPAM expert panel described below. The elaborated SPAM conceptual framework is shown in Fig. 1. It encompasses three main domains (structure, process and outcome) and twelve sections separated into accessibility of care and content of care (access to the PC system, funding of PC, organization of resources, Medical/Paramedical training, workflow of resources, management of knowledge, clinical- and interpersonal care, health status, satisfaction of consumers/ PC providers and equity).

### Definition of primary care in Switzerland

In Switzerland and internationally there is no existing consensus for the definition of a PC physician. After literature search [15–21] and approval by the SPAM expert panel, the following operational definition was set**:** a PC physician (Médecins de premier recours/ Ärzte in der Grundversorgung) is a physician with at least one of the following title from the Swiss Medical Association (FMH = Foederatio Medicorum Helveticorum) title: General Medicine (before 2011) or Internal Medicine (before 2011) or General Internal Medicine (since 2011) or Medical Practitioner (=Médecin praticien / praticienne; praktischer Arzt). Family physician and general practitioner (GP), in this project, refer to the same definition of PC physician. It is also true that PC (and by extension Primary Health Care) is not exclusively based on physicians but includes also other health professionals such as medical assistants, nurses or occupational therapists. These professionals might work in PC practices or in others community structures. In Switzerland most PC practices consists however mostly of GP’s and medical assistants, and very few other professionals. This explains why we decided, in a first step, to focus mostly PC physicians. But it is anticipated to expand in the future the monitoring to other professions working in PC.

### Selection of indicators

Based on the review of existing models, we made a selection of a set of indicators. An additional scoping literature review was performed in order to complete the selection of indicators in domains not sufficiently covered by PHAMEU such as prevention, medical education, or equity.

The methodology of this study, a consensus semi-qualitative RAND method has indeed been chosen in the specific context of the Swiss setting. But this is not specific to the development of indicators in Switzerland. Indeed, consensus approaches are widely recognized as the method of choice to select indicators in complex fields such as health services research [22]. This is especially true in Switzerland where consensus on the governance of the health system is difficult to obtain. Indeed, as it was mentioned, the health system is highly fragmented with an important independence of actors, mixing private and public sectors. As an example, it can be mentioned that health insurances plans are compulsory for the population but are provided by more than forty private non-profit, largely unregulated, companies. In such a context, it is hardly impossible to reach large consensus for implementing nationwide monitoring system of the health system. This explains why we decided to set-up the SPAM program based on a panel of experts representing all major actors in PC.

Following the RAND appropriateness methodology, we selected experts of major institutions involved in PC in Switzerland (Additional file 1). We conducted first of all two rounds of a modified RAND process with 24 national and international experts to evaluate the 365 selected indicators for their validity and clarity. In a 1st rating round, they were asked to vote individually on a 9-point scale in order to assess validity and clarity of all indicators. Validity was defined as “*the extent to which the indicator accurately represent the concept being assessed and is an appropriate measurement of the functioning and performance of the Swiss PC system”* (Scale: 1 = invalid, 5 = inexplicit, 9 = highly valid). Clarity was defined as *“the indicator clearly named (non-ambiguous)”,* (Scale: 1 = not at all clear, 5 = ambiguous, 9 = very clear). The second round was an interactive face-to-face panel meeting with all SPAM experts re-rating on all criteria. Indicators with high rated validity, clarity and agreement were classified as primary indicators, those which uncertain validity or clarity as secondary indicators. Primary or secondary indicators were stratified in sub-indicators where necessary, e.g. if sub-items of an indicator should be monitored e.g. patient-doctor travel distances.

We conducted a third round of ratings, with three objectives: 1) Global validation of structure and the indicators’ reformulation and selection after the second round. 2) Selecting priority indicators to provide a manageable amount of indicators to perform regular monitoring and conduct regular update reports of Swiss PC (every 1–3 years). 3) Identification for each of twelve sections of one to five priority indicators, with the rule that the number of priority indicators retained is proportionate to the total number of primary indicators by section.

For the third round, SPAM experts (same Expert Panel in the three rounds) were informed of the results of the first two rounds by e-mail and were asked to vote via a web-based survey, oriented on a modified RAND-process [23–25]. The vote took place from October 2013 till March 2014 online via SurveyMonkey®. Groups of primary indicators were assessed along two steps: 1) individual importance [Scale: 1 = essential to have (as a priority indicator); 2 = important to have (as a priority indicator); 3 = nice to have (as a priority indicator); 4 = might be good to have (as a priority indicator); 5 = not very important to have (as a priority indicator)]; 2) priorization for a basic assessment of the Swiss PC system’s performance and effectiveness. The final selection (choice of a limited number of priority indicators) was based on medians and Interquartile range analyzed with STATA® 13.

## Results

**Fig. 2:**
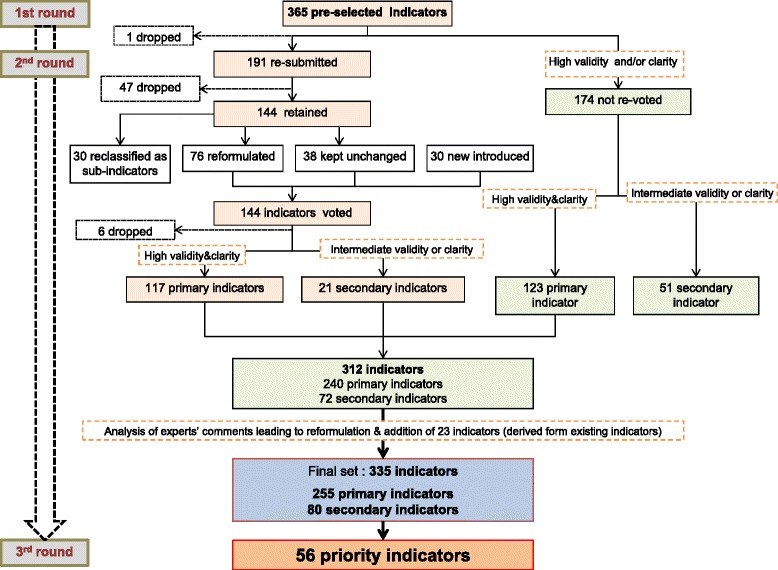
Selection of indicators for the SPAM program

Based on the literature search, 365 indicators were preselected. Indicators from the sections “access to the PC system”, “governance and “funding”, “workflow and resources”, “clinical care” and health status” and “consumer’s satisfaction” have been mainly established based on the PHAMEU indicators. Concerning prevention, we used indicators published by MacColl et al. [26]. A number of indicators have been added in the field of equity, used in the project QUALICOPC (Quality and Costs of Primary Care in Europe) [27] and the studies and publications of R. Rudd et coll. (School of Public Health, Harvard) [28, 29].The sections “Management of Knowledge” and “Satisfaction of providers” are currently not covered with indicators; their development will be subject of further investigations. The SPAM experts (96% participation) voted during the 1st round. 212 indicators (58%) were considered as valid and 346 (95%) clear by SPAM experts; 153 (42%) of uncertain validity, 19 (5%) uncertain clarity and one was voted invalid and was dropped. The SPAM experts agreed in 82.5% of the votes. One hundred ninety one indicators were resubmitted for discussion to the second round. According to written comments of experts along the first round, some indicators were reformulated for discussion in the second round. Indicators which have not been submitted in the second round were kept as primary indicators if the rating in validity and clarity was ≥7 or as secondary indicator if rated 4–6 in validity and/or clarity (Fig. 2). If more than 1/3 of the experts’ answers were in the extreme ranges of the scale, the indicator was considered with uncertain validity or was modified e.g. as not all the indicators were submitted for the second round as we decided not to resubmit indicators with high level of agreement, validity and clarity. Following the 2nd round, 47 indicators were dropped, 76 reformulated, 30 new indicators were introduced in the SPAM experts voting booklet and 30 were considered as sub indicator. The revised indicators were submitted for revoting by the SPAM expert Panel. (participation rate of 67%). 135 indicators (77.6%) have been rated as valid and 169 indicators (97.1%) as clear. Agreement was meeting with 61.5%.

Finally, 255 indicators were kept for the program as primary and 80 as secondary indicators **(**Fig. 2). The indicator adjustment process resulted after the 2nd round in a higher number of indicators that have initially been selected during the SPAM expert meeting. This was related to their demands of specification and harmonisation of the indicators, e.g. same medical specialties to be equally represented coherent groups across the instrument.

**Fig. 3:**
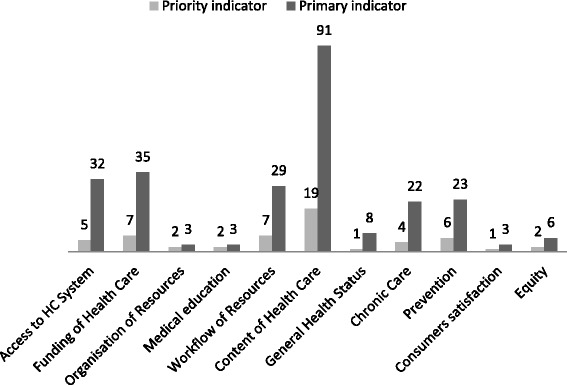
Total number of primary to Priority indicators by section

Two hundred fifty five primary indicators were submitted to the third round. The SPAM experts (79% participation) voted in two steps 255 indicators (95% completed surveys). For technical reasons (no votes registered) a supplementary round was done for 6 indicators. A total of 94 indicators were chosen by the SPAM experts. The median rating of importance of all indicators was 2. Finally, 56 priority indicators of all domains **(**Fig. 3) were retained and are displayed in Table 1.

**Table 1:** 56 Priority Indicators of the SPAM program (*number in italic* = indicator’s number)

Domain		Chapter	Section
A Structure		1. Accessibility	1.1. Access to the Health care System
1.1.1 Development of workforce supply			
*1*	% by which the density of GP / FAMILY PHYSICIANS has increased or reduced over the most recent available 5 year period		
1.1.2 Density available PC workforce			
*2*	Density of GP / FAMILY PHYSICIANS per 100′000 population		
1.1.3 GP-specialist ratio			
*3*	Ratio of active GPs/active medical specialists		
1.1.4 Age distribution GPs			
*4*	Median age of practicing GPs on NATIONAL LEVEL		
1.1.5 Social Accessibility			
*5*	Average time for patients to travel from their home to their GPs independently of the mean of transport by RURAL AREAS		
	Priority sub indicators		
	1	Average time for patients to travel from their home to their GPs independently of the mean of transport by RURAL AREAS ≤20 min	
	2	Average time for patients to travel from their home to their GPs independently of the mean of transport by RURAL AREAS ≥21 to 40 min	
	3	Average time for patients to travel from their home to their GPs independently of the mean of transport by RURAL AREAS ≥41 to 60 min	
	4	Average time for patients to travel from their home to their GPs independently of the mean of transport by RURAL AREAS ≥61 min	
A Structure		1. Accessibility	1.1. Funding of Health Care
1.2.1 Total PC expenditure			
*6*	Ratio of total expenditure on PC / total expenditure on health		
1.2.2 Expenditure on prevention and public health			
*7*	Ratio of total expenditure on prevention and public health / total expenditure on health		
1.2.3 Employment status of GPs			
*8*	% of practicing GPs that are salaried by an INTEGRATED CARE ORGANIZATION		
1.2.4 Financial status of GPs compared to a specialist			
*9*	Ratio of annual median income of a GP to the annual median income of a SPECIALIST		
1.2.5 Income of GPs			
*10*	Annual median income of a GP on NATIONAL level		
1.2.6 Cost-sharing for GP care			
*11*	% of patients co-payment (next to coverage by insurance) for visit to GP as a ratio of total cost for visit to the GP		
1.2.7 Medical insurances			
*12*	% of patients with complementary insurance		
A Structure		1. Accessibility	1.1. Organisation of Resources
1.3.1 Gate keeping System			
*13*	General indicator: % of patients with “GP models” insurance contracts		
*14*	General indicator: % of patients accessing other disciplines without referral of GP		
A Structure		2 Health care	2.1 Medical education
2.1.1 Medical graduate trained in family medicine			
*15*	Number of FMH titles in GIM obtained as ratio of the total number of FMH titles per year		
2.1.2 New family medicine practices			
*16*	Number of FMH-GIM doctors starting their activity in a private practice as a ratio of the total number of doctors with FMH-titles starting their activity in a private practice per year		
B Output		3 Workflow of Resources	
3.1. Workload of GPs			
*17*	Average number of working hours per week of GPs		
3.2 Medical record keeping			
*18*	% of GPs keeping (or reporting to keep) electronic clinical records for all patient contacts routinely		
3.3 Specialist-GP communication			
*19*	% of specialists communication back to referring GP after an episode of treatment		
3.4 Shared practice			
*20*	% of PC practices that are single handed (solo) as a ratio of all practices		
*21*	% of PC practices with mixed practice with GPs and medical specialists		
3.5 Duration of GP consultation			
*22*	Average consultation length (in minutes) of GPs		
3.6 GP consultations			
*23*	Number of GP consultations per capita per year		
B Output		4. Content of Health Care	4.1 Clinical Care
4.1.1 Medical equipment available			
*24*	% of practices having the following equipment in PC facilities: RADIOLOGY EQUIPMENT (X-Ray)		
*25*	% of practices having the following equipment in PC facilities: LABORATORY		
*26*	% of practices having the following equipment in PC facilities: DRUG DISPENSARY		
*27*	% of practices having the following equipment in PC facilities: ECG		
4.1.2 First contact care			
*28*	% of GP providing first contact care for WOMAN AGED 35 WITH PSYCHOSOCIAL PROBLEMS		
*29*	% of GP providing first contact care for patient with ALCOHOL ADDICTION PROBLEMS		
4.1.3 Treatment and follow-up of diseases			
*30*	% of GP’s providing treatment/follow-up care for patients with LOWER BACK PAIN		
*31*	% of GP’s providing treatment/follow-up care for patients with MILD DEPRESSION		
*32*	% of GP’s providing treatment/follow-up care for patients ADMITTED TO A NURSING HOME / CONVALESCENT HOME		
4.1.4 Medical technical procedures			
*33*	% of GP providing: WOUND SUTURING		
4.1.5 GP contacts without referral			
*34*	% of total patient contacts handled solely by GPs without referrals to other providers		
4.1.6 Health promotion			
*35*	% of GPs who offer individual counselling to the practice population. Counselling in case of OBESITY		
*36*	% of GPs who offer individual counselling to the practice population. Counselling in case of SMOKING CESSATION		
*37*	% of GPs who offer individual counselling to the practice population. Counselling in case of PROBLEMATIC ALCOHOL CONSUMPTION		
4.1.7 Preventive care			
*38*	% of GPs providing: SKIN SCREENING (FOR SKIN CANCER)		
*39*	% of GPs providing: INFLUENZA VACCINATION FOR HIGH-RISK GROUPS		
*40*	% of GPs providing: BLOOD SUGAR CONTROL		
*41*	% of GPs providing: WEIGHT CONTROL		
*42*	% of GPs providing: CHOLESTEROL LEVEL CONTROL		
C Outcome		5 Status of patient’s health	5.1. General
5.1.1 Antibiotics consumption			
*43*	Defined daily doses of antibiotics use in ambulatory care per 1000 inhabitants per day		
C Outcome		5 Status of patient’s health	5.2 Chronic Care
5.2.1 Diabetes care			
*44*	% of diabetic patients aged >25 years with overweight and obesity and BMI measured in the last 12 months		
5.2.2 COPD care			
*45*	% of patients with COPD that have had a follow-up visit in primary care during the last year		
5.2.3 Control of hypertension			
*46*	% of patients identified as hypertensive whose BP recorded in past year		
5.2.4 Use of angiotensin converting enzyme inhibitors in those with heart failure			
*47*	% of patients with heart failure who have a prescription for ACE inhibitors		
C Outcome		5 Status of patient’s health	5.3 Prevention
5.3.1 Influenza vaccination in those aged over 65 years			
*48*	% patients aged 65+ vaccinated against flu		
5.3.2 Breast cancer screening			
*49*	% of women aged 52–69 yrs. who had at least 1 mammogram in the past 3 yrs		
5.3.3 Cervical cancer screening			
*50*	% of women aged 21–64 yrs. who had at least 1 Pap test in the past 3 yrs		
5.3.4 Aspirin for patients at high risk of coronary or ischemic cerebrovascular events			
*51*	% of patients with diagnosis of IHD who take aspirin		
5.3.5 Smoking cessation			
*52*	% of patients whose smoking status recorded		
*53*	% of patients who are current smokers and have received advice on stopping smoking or nicotine replacement therapy		
C Outcome		6. Consumers satisfaction	6.1 Patients satisfaction
6.1.1 Patient satisfaction with the GP (PDRQ-9)			
	*54*	% of patients who are satisfied with their relation with their GP/PC physician	
		Priority sub indicators	
	5	% of patients assessing that their GP is helping them	
	6	% of patients assessing that their GP is dedicated to help them	
	7	% of patients assessing that their GP has enough time for them	
	8	% of patients have confidence in their GP	
	9	% of patients assessing that their GP understands them	
	10	% of patients assessing that they agree with their GP on the nature of my medical symptoms	
	11	% of patients assessing that they can talk to their GP	
	12	% of patients that feel content with their GP’s treatment	
	13	% of patients assessing that their GP is easily accessible	
C Outcome		7. Equity	7.1 Access
7.1.1 Restriction of access to GP			
*55*		% of patient who postponed or abstained from a visit to his doctor or another GP when it was needed in the past 12 months	
		7.1.2 Psychological needs asked by GP	
*56*		% of GP practices having elaborated and/ or adopted procedures to meet the psycho-social needs of individual patients	

## Discussion

A conceptual framework was developed, for selecting PC indicators in three main domains (structure, process, outcome) and twelve sections (accessibility, financing, workflow and organisation of resources; medical/paramedical training, clinical/interpersonal care; health status, consumer- / PC providers satisfaction and equity) for a continuous monitoring of PC in Switzerland. Through a modified RAND process 255 primary & 80 secondary indicators have been selected and 56 were retained as priority indicators that will serve as a basis for an ongoing monitoring of the Swiss PC system.

This monitoring tool has been tailored for the Swiss Primary Care system. Its elaboration was conducted independently, but it is endorsed by the main institutions active in primary care in Switzerland, which is one of its main strength. The explicit formulation of the indicators and their formulation based on a review of international monitoring instruments facilitate reproducibility and international comparisons. A limitation of the instrument might be that not yet all subsections of the conceptual framework could be included in the indicator selection process but the intention was to built a robust instrument step by step with indicators of good quality and to avoid the presentation of a an exhaustive but premature construction.

We are also aware of the limitations of the adapted RAND method as it is mainly based on expert opinion. We believe however, that the RAND method remains in this specific context the most adequate approach, especially in Switzerland where consensus among health actors is difficult to achieve due to the fragmentation of the health system and the complex blending of private and public sectors.

On the other hand, the process of development of this monitoring tool is also quiet similar to De Vellis’ work and the present study corresponds to step 1 to 5 (out of 8) of its described method [30]. In brief, step 1 “Determine clearly what you want to measure” was done in our “Objective of the present study”-section. Step 2 “Generate in item pool” was the “selection of indicators” – section. Step 3 “Determine a format of measurement” was performed along the Obsan report published in 2016 [31], in defining exactly the formulas used to specify the indicators. Step 4 “Have initial item pool reviewed by experts” corresponds also to the “Selection of indicators” part of the present work. Finally, the modified-RAND process refers to Step 5 of deVallis “Consider Inclusion of Validated items”. Not presented here, step 6: “Administer items to a development Sample” was performed along a national report on the functioning of PC in Switzerland. We identified via existing literature the data related to the indicators and the indicators were precisely defined in formulas with numerators and denominators. Missing data in existing data sources were partially generated using an ad hoc sampling of PC practices (the SPAM PC-physician network) [32]. Step 7 “Evaluate the items” will be the next step. We will indeed analyze critically existing indicators as well as data source availability. Finally Step 8 “Optimize scale length” will be performed by collaborating with health authorities in order to improve sustainability of the monitoring tool and widen the data sample for the indicators and delivering information to the actors.

### SPAM and PHAMEU

As mentioned in the introduction, the PHAMEU tool was judged insufficient to assess the Swiss PC system. First, a generic tool such as PHAMEU might be useful to draw general comparisons between countries but is unable to capture more fine changes within countries. In that regard, a recent study comparing PHAMEU indicators for France and Switzerland end up with very few comparable indicators [33]. Second, the choice of the indicators of the PHAMEU tool insufficiently captures contextual factors linked to the highly bismarckian Swiss health system (ie based on private health insurances with limited state regulation). Last, the formulation of indicators is often too vague to guide accurately the construction of the indicators. All these limitations were at least partly overcome in the SPAM tool by redefining some indictors for the Swiss context and unequivocal definitions for the indictors were set.

### Contextualization of the selection of the 56 indicators

It is also of interest to briefly outline how the SPAM framework might have been influenced by the local and historical context of the Swiss Health system, referring to the path dependency theory. Without going into details, this theory postulates that behaviors, in that case of PC actors and by extension the SPAM expert panel, are influenced by the history of the institutions they belong to [34]. First, we observe that the SPAM monitoring tool is a kind of compromise between the needs to introduce some level of organizational monitoring of PC structures (for several reasons, but probably, and among other factors, because of the high cost of the system and the anticipated shortage of GP’s) and to preserve the traditional liberal private practice. Second, it is interesting to note, as mentioned in the methodological part, that the present selection of indicators focuses mostly of PC practices and physicians, which reflects the long history of a PC system that is based mostly on the work of solo physicians “doing everything”. Most experts of the panel judging, maybe partly unconsciously, that the way it is currently organized will not change. It is important to be aware of that, for the future development of the monitoring tool, as the present selection of indicators as limited value to capture changes in regards, for example, to new models of PC that include more interprofessonality. A more in-depth analysis, from a sociological perspective, might be very interesting to explain how the selection of indicators was made, but is out of the scoop of the present paper.

### Future developments

As mentioned in the introduction, it is traditionally believed that the access and availability of data concerning the PC sector is limited in Switzerland. The anticipated lack of available data to inform the indicators might be a further challenge in the upcoming data collection process.

For example, nationwide surveys an ad hoc prospective data collection through the SPAM network of PC physicians might be used. This is a representative practice-base research network (PBRN) of more than 200 PC physicians that was created specifically for the SPAM program in parallel to the QUALICOPC study and is dedicated to the evaluation of the PC system [4, 35]. As no routine data collection system is in place in Switzerland (only half of PC physicians have electronic medical records which are mostly not interoperable), [36] it will be essential to be able to access high quality data that might accurately reflect the overall Swiss PC system. However, the implementation of routinely used electronic medical records and PC information systems presents an important financial investment raising the question of another supplementary administrative task for the health professionals and the need of an appropriate remuneration scheme including incentives to nurture and maintain this instrument. In that perspective, a federal project launched in 2015 (“MARS” in the policy “Health 2020”) aims to routinely collect data about medical ambulatory sector, mainly focusing on organizational aspects.

Practically, a fact-sheet is generated for each priority indicator. It specifies the descriptive definition of the indicators, results (data source, quality of the data source, previous findings, indicator rational), method of calculation (formula, numerator, denominator), stratification, indicator subcategories, Interpretation and references. This is not presented here, but was achieved in a report published by the Swiss health observatory (http://www.obsan.ch), with most indicators being filled [31]. In the future, priority indicators will be updated regularly and, theme-oriented specific surveys that will use the entire set of primary and secondary indicators will be conducted in parallel to the main monitoring.

Linkage between different indicators is also anticipated in order to provide a more accurate measure of the performance of the PC system (e.g. relate the social accessibility of PC physicians to the outcome of perceived restrictions to access). The Obsan’s report on the functioning of PC in Switzerland might also help to establish priorities for improvement but doesn’t aim to set achievement targets. Indeed, targets won’t be set for several reasons. First, this project aims at providing a global description of the functioning of the PC system and not to provide individual performance measurements. Second, we estimate that insufficient information over time is available to establish targets at this point. Last, instead of setting arbitrary targets, comparison of the indicator results with the data of other international monitoring instruments might provide a more insightful knowledge of the overall performance of the PC Swiss system. The initial monitoring tool focuses mainly on PC physicians and their organisation, but it is already planned to extend the indicators to other professionals in practices (nurses, medical assistants,…). This is already anticipated in the presented framework under the “workflow of resources” domain (collaboration and interprofessionnality).

### Policy perspective

From a policy perspective, the setting up a monitoring tool should not focus only on the creation of indicators and collection of data. Indeed, probably the most challenging part is that it could allow a process of change (at policy level as well as at practice level). The acceptability of the tool plays thus s crucial role and is considered as a key element of indicators’ development [22]. Indeed a monitoring instrument might be able to support health policy adjustments and changes in the organization and delivery of PC only if all actors agree on the meaningfulness and accuracy of the indicators or monitoring tool. In that sense, as a first step, adopting a RAND consensus group with most important actors can help to make it more acceptable. But if one would like to make changes really occurring, important efforts should be made to assess the acceptability of the tool. At practice level, for example, PC physicians of the SPAM network might be interested to compare their performance of health care delivery with peers and further consensus on practice standards might be set. This is out of the scoop of the present work to explore the acceptability of the tool, but this will need to be addressed when it will be implemented. A qualitative study is already planned to address this issue and to see how policy makers and PC actors would be willing to work with this tool.

## Conclusions

The development of the SPAM program allowed the construction of a consensual instrument in a traditionally unregulated health system by a modified RAND process. Moreover, its 56 priority indicators, which were selected through a modified RAND process, make the SPAM instrument a comprehensive tool, able to better understand the PC system’s performance and effectiveness as well as to identify potential ways to improve quality of care. It was also designed to allow international comparison; therefore indicators selected for the SPAM program might also be used and adpated in other countries desiring to monitor their own PC system. The strength of implementing such monitoring instrument is to make the best use of available data in combination with specific prospective data collection. Further challenges will be to regularly update indicators and to assess its acceptability, validity and sensitivity-to-change over time.

## Additional file

Additional file 1: Composition of the SPAM expert Panel group. (DOCX 12 kb)

## Abbreviations

AQUIK: Ambulatory Quality indicators and figures (Germany)
CDS: Swiss Conference of the cantonal health directors
CMPR: College of Primary Care Medicine
FMH: Swiss Medical Association
GDP: Gross domestic product
IHAMB: Institute of Family Medicine, University Basel
IRDES: Institute for research and information in health economics (Paris)
IUFRS: Institute for education and research in health care, University of Lausanne
IUMG: Institute of General Medicine, University of Lausanne
IUMSP: Institute of social and preventive Medicine, University of Lausanne
MFE: Swiss Union of the Family Practitioners and Pediatricians
NIVEL: Netherlands Institute for Health Service Research
OBSAN: Swiss Health Observatory
OFS: Federal Office of statistics
OFSP: Federal Office of Public Health
PC: Primary Care
PHAMEU: Primary Health Care Activity Monitor for Europe
QOF: Quality and Outcomes Framework
QUALICOPC: Quality and Costs of Primary Care in Europe
SPAM: Swiss Primary Care Active Monitoring
SSMI: Swiss Society of General Internal Medicine
Swiss TPH: Swiss Tropical and Public Health Institute
