# Supplementary material for: Development of a monitoring instrument to assess the performance of the Swiss primary care system
Source: BMC Health Serv Res. 2017 Nov 29;17:789. doi: 10.1186/s12913-017-2696-z (PMC5707782; doi:10.1186/s12913-017-2696-z)
Supplement: Additional file 1: — Composition of the SPAM expert Panel group. (DOCX 12 kb) [file 12913_2017_2696_MOESM1_ESM.docx]

**Additional file 1 : Composition of the SPAM expert Panel group**

Federal Office of Public Health (BAG), Swiss Medical Association (FMH), Swiss Health Observatory (Obsan), Swiss Health Care Insurers (SantéSuisse), College of primary Care Medicine (KHM), Association of General Practitioners and Paediatrician Switzerland (MFE), Swiss Society of General Internal Medicine (SGIM), Institute of social and preventive medicine (IUMSP), Institute of general medicine Basel (IHAMB), Swiss Tropical and Public Health Institute (Swiss TPH), Institute of formation and research in health care (IUFRS) and the Swiss conference of cantonal health care directors (GDK). One member was an international expert representing the “Institut National de Santé Publique du Québec”.
